# An exploration of expectations and perceptions of practicing physicians on the implementation of computerized clinical decision support systems using a Qsort approach

**DOI:** 10.1186/s12911-022-01933-3

**Published:** 2022-07-16

**Authors:** Wim Van Biesen, Daan Van Cauwenberge, Johan Decruyenaere, Tamara Leune, Sigrid Sterckx

**Affiliations:** 1 Department of Nephrology, Ghent University Hospital, Corneel Heymanslaan 10, 0K12IA, Ghent, Belgium; 2 Consortium for Justifiable Digital Healthcare, Ghent University Hospital, Ghent, Belgium; 3 Department of Philosophy and Moral Sciences, Bioethics Institute Ghent, Ghent University, Ghent, Belgium; 4 Department of Intensive Care Medicine, Ghent University Hospital, Ghent, Belgium

**Keywords:** Big data, Clinical decision support, Medicine, Artificial intelligence

## Abstract

**Background:**

There is increasing interest in incorporating clinical decision support (CDS) into electronic healthcare records (EHR). Successful implementation of CDS systems depends on acceptance of them by healthcare workers. We used a mix of quantitative and qualitative methods starting from Qsort methodology to explore expectations and perceptions of practicing physicians on the use of CDS incorporated in EHR.

**Methods:**

The study was performed in a large tertiary care academic hospital. We used a mixed approach with a Q-sort based classification of pre-defined reactions to clinical case vignettes combined with a thinking-aloud approach, taking into account COREQ recommendations The open source software of Ken-Q Analysis version 1.0.6. was used for the quantitative analysis, using principal components and a Varimax rotation. For the qualitative analysis, a thematic analysis based on the four main themes was performed based on the audiotapes and field notes.

**Results:**

Thirty physicians were interviewed (7 in training, 8 junior staff and 15 senior staff; 16 females). Nearly all respondents were strongly averse towards interruptive messages, especially when these also were obstructive. Obstructive interruption was considered to be acceptable only when it increases safety, is adjustable to user expertise level and/or allows deviations when the end-user explains why a deviation is desirable in the case at issue. Transparency was deemed an essential feature, which seems to boil down to providing sufficient clarification on the factors underlying the recommendations of the CDS, so that these can be compared against the physicians’ existing knowledge, beliefs and convictions.

**Conclusion:**

Avoidance of disruptive workflows and transparency of the underlying decision processes are important points to consider when developing CDS systems incorporated in EHR.

**Supplementary Information:**

The online version contains supplementary material available at 10.1186/s12911-022-01933-3.

## Introduction

Diagnosing and managing patients’ conditions are key factors of patient care [1]. Interest in application of Computerized clinical decision support systems (CDSS) fueled by artificial intelligence (AI) is progressively gaining momentum. Whilst external validation is still lacking [2–5], expectations of how implementation of AI-based CDSS can improve health care are high [6]. In literature, CDSS is mostly defined as a computer-based system intended to support clinical decision making in everyday patient care by presenting to the healthcare worker an integrated summary of clinically relevant patient information. The emergence of automated CDSS is facilitated by the introduction of electronic health records (EHRs) and Computerized Provider Order Entry systems (CPOE). However, the introduction of EHR/CPOE has also been linked to increased incidence of physician burnout and decreased patient satisfaction. These observations have been attributed to the enhanced additional administrative burden induced by EHR/CPOE (“death by a thousand clicks” [7]), and the feeling that computers obstruct true involvement between physician and patient [8] whilst they do not improve clinically relevant outcomes [9]. As a consequence, some perceive the implementation of CDSS into everyday care a major step forward [6], whereas others perceive it rather as a threat [10].

The success of the implementation and performance of CDSS depends on technical characteristics of the software, the clinical aspects of the task at hand and the expertise of the physician with the CDSS. Next to these, a substantial human factor remains, and acceptance of the CDSS is essential. Many interruptive medication alerts are e.g. simply ignored by the operator [11–13]. In addition, the problem of alert fatigue is a well-established downside of interruptive messaging in CDSS [5, 14]. Different aspects of successful implementation of CDS devices have been explored, however mostly in narrow contexts for well-defined and delineated clinical problems [4, 5]. Little evidence is available on which factors should be taken into account to maximize uptake by clinicians when incorporating CDSS in to general EHRs/CPOEs [15, 16]. Most EHRs/CPOEs available on the market today are designed from an administrative and informatics perspective [8]. They rarely consider the specific requirements of clinical tasks [17]. Most systems do not take into account local conditions and culture, and most offer general solutions for general problems, rather than specific solutions for the actual problems the clinicians and their patients are facing [18, 19]. As a consequence, they produce unrealistic, inapt or plainly unsuitable advice for the local setting. Therefore, there is a huge gap between what health care workers have to put in to the system to make it work, mainly administrative information, and what they get out in terms of improved care for their patients.

We hypothesized that this friction causes frustration and decreases the probability of successful implementation of CDSS in an EHR/CPOE. On the other hand, overconfidence in the computerized CDSS might also occur even when the advice is misleading [20, 21].

Therefore, we designed a study with a mix of a quantitative and a qualitative method to thematically explore the reactions and underlying reasoning of physicians when confronted with vignettes in which hypothetical CDSS incorporated in an EHR were presented.

## Methods

The study was performed in an academic hospital in a transition to selecting, customizing and implementing a new EHR/CPOE system. The current EHR/CPOE has been in use for more than 10 years, but does not incorporate possibilities for CDSS. All participants were thus familiar with the concept of EHR/CPOE, however, their exposure to CDSS incorporated in EHR/CPOE was low.

We used an approach with a Q-sort based [22] classification of pre-defined reactions to clinical case vignettes (Additional file 1: appendix 1), in combination with a thinking-aloud approach in which reasoning and attitudes of the participant during the classification task were solicited. All sessions were done by the same interviewer (WVB) with expertise in the field of computerized CDSS. All sessions were audiotaped and typed out verbatim afterwards. During the whole process, we adhered strictly to COREQ recommendations [23].

Based on literature and consensus by the research team, a concourse of statements was created. These statements described potential actions of a (hypothetical) CDSS in four well defined clinical settings (Additional file 1: appendix 1). We opted for a structured approach based on a prior hypotheses that acceptance of clinical decision support by clinicians is influenced 1/by the transparency of the CDSS; 2/ the degree of certainty regarding the advice provided by the CDSS; 3/ interruption of workflow with or without obstructivity; 4/ the type of problem at hand. None of the vignettes explicitly mentioned whether the CDSS relied on a rule based system or a self-learning system.

We developed thirty statements [24], distributed over four clinical case vignettes, varied in four different attributes [25]:

1. *Transparency of the support* how well is the reasoning of the device explained to the user?
2. *Ways of expressing (un)certainty of the system on the correctness of its advice* absolute truth and certainty are rare in medical conditions. Therefore, it is essential that a CDSS can express this uncertainty in its advice. Different ways and gradations of expressing this uncertainty were introduced in the vignettes.
3. *Interruption of workflow with or without obstruction* CDSS can produce advice on request, but also in an unsolicited (automated) fashion while working with the system. This can *interrupt* the workflow, meaning the user is distracted from her activity and needs to perform an unplanned action. This interruption can be non-obstructive (workflow can go on as planned e.g. by pressing an “ignore” button), or be *obstructive* (planned workflow can only go on after the unplanned action has actually been performed or planned workflow needs to be aborted).
4. *Type of problem* CDSS can be used for easy or more complex tasks.

In a pilot, statements were evaluated for clarity and lack of ambiguity (Additional file 2).

In the first two vignettes, the focus was on decision support for medication orders, covering formulary, drug-drug interaction and drug-condition alerts [26]. In the third vignette, a diagnostic problem was raised, assessing automated CDSS for order entry as well as for presenting and interpreting results of the ordered diagnostic tests. In the last vignette, a more advanced CDSS for handling a complex clinical problem was presented (Additional file 3).

We intended to interview 30 physicians, with purposive sampling to achieve a mix of gender and level of expertise (trainee, junior staff, senior staff) [22, 25].

First, a list of 30 physicians consenting to take part was created on a first come first served basis to avoid selection bias. Next, a random allocation of order for interviewing was created. Respondents were interviewed in a silent room, and all sources of distraction were avoided as much as possible. All interviews took place in the period July–August 2021 (Additional file 4).

### Statistics and analysis

For the quantitative analysis of the Q-sort, we used the open source software of Ken-Q Analysis version 1.0.6 (https://shawnbanasick.github.io/ken-q-analysis) [27]. For each of the four vignettes, principal components were extracted, and a Varimax rotation on the factors with an Eigenvalue > 1.5 was performed subsequently as it was deemed there was no theoretical justification for a judgmental rotation, and we intended to reveal the range of viewpoints favored by our participants [28].

For the qualitative analysis, a thematic extraction was performed using NVIVO software based on the audiotapes of the thinking-aloud during the interviews. Themes and concepts were grouped and re-grouped until all concepts were placed under a non-overlapping header conform the hypothesized four attributes. This was first done in two groups (WVB and TL in group one and SS and DVC in group two) separately, and was then triangulated with the entire research team in two discussion sessions. After this, SS and DVC checked all the audiotaped interviews again to screen for fit of our thematic analysis with what was actually conveyed during the interviews, and to detect any missing viewpoints. The results and interpretation of the thematic analysis were discussed at length with a small group of interested peers at two different occasions. These readback groups did not include all the original respondents, and also contained participants who did not participate in the interviews (Additional file 5).

## Results

As planned, 30 physicians were interviewed, 7 in training (IT), 8 junior staff (JS) and 15 senior staff (SS); 16 females.

### Q-sort: quantitative results

*Vignette 1 (Additional file **1**: appendix 1)*: Unrotated factor analysis yielded four factors, explaining 91% of variance (66%, 13%, 7% and 5% resp.). Factor one loaded on to fifteen respondents (6 male, 10SS, 2JS and 3IT). Obstructive interruption was scored as a strongly negative element by all participants, and was the major determinant of this factor. Suggesting an alternative solution by the CDSS consistently mitigated this negative attitude. Factor two loaded on six respondents (3 male, 2IT, 2JS, 1SS). Also in this factor, obstructive interruption ranked high. This was mitigated by transparency and pragmatic suggestions by the CDSS, making it different from factor 1. Factor 3 loaded on five respondents (2 female, 3SS, 2JS) and was also mainly driven by interruption, that could however be overruled. Factor 4 loaded only to one person (male trainee) although it explained 7% of total variance. With a negative attitude towards obstructivity, it also highlighted a preference for absolute advice rather than (soft) suggestions. One statement (1A, Additional file 1: appendix 1) consistently ranked lowest (consensus statement, rank -2, Z-score variance 0.0007) in all 4 factors. This statement was obstructive, and it did not provide transparency. Another obstructive statement without transparency nor certainty on the advice (1H) was not scored positively in any of the factors.

*Vignette 2* Unrotated factor analysis yielded four factors explaining 92% of variance (43%, 24%, 14% and 13% resp). Factor 1 loaded on to seven respondents (4 male, 2SS, 4JS and 1IT). Obstructive interruption was scored strongly negative by all participants. However, when this obstruction served to increase safety (statements 2B and 2F), it was scored very positively. Factor 2 loaded on only three respondents (2 male, 1IT, 2SS). Within this factor, obstructive interruption ranked high, and this was not overruled by safety concerns. Automated presentation of results was positively appreciated, especially when presented on a smartphone. Factor 3 loaded only on one respondent, despite explaining 18% of variance (1 female, SS). It was not driven by obstructive interruption, regardless of safety concerns. Suggestions made by the CDSS were appreciated, unless presented on a smartphone. Factor 4 loaded to five respondents (3 male, 3SS, 1JS and 1IT). Besides a negative attitude towards obstructive interruption, automated presentation of results was highly appreciated when presented as a pop-up in the EHR, but not on a smartphone. Within this vignette, one statement (2A) ranked lowest in 3 out of 4 factors (rank -2, Z score variance 0.31). This statement was highly obstructive, and did not provide any transparency. Another statement (2H) was not scored negatively by any of the factors. This statement was helpful, transparent and enhanced safety.

*Vignette 3* Unrotated factor analysis yielded four factors explaining 97% of variance (58%, 17%, 12% and 10% resp.). Factor 1 loaded on to eight respondents (5 male, 4SS, 3JS and 1IT). Obstructive interruption was scored strongly negative by all participants. Adding transparency to help understand the advice was appreciated by all respondents. Factor 2 loaded on eight respondents (4 male, 6IT, 2SS), and was thus highly driven by physicians in training, all rating obstructive interruption highly negative. Respondents all highly appreciated CDSS suggestions when presented in a non-obstructive manner.

Factor 3 loaded only on two respondents, but explained 12% of variance (1 female, 2SS). It strongly preferred safety over obstructivity (statements 3B, 3C, and 3D). Factor 4 even loaded only to one respondent (female, SS), but explained 15% of variance. It expressed a negative attitude towards obstructive interruption. Statements 3E and 3F did not get any negative ranking in any of the 4 factors. These statements were not obstructive, provided transparency and some degree of AI like advice. Statements 3A and 3D with an explicit obstructive character did not get any positive ranking in any of the factors.

*Vignette 4.* Unrotated factor analysis yielded four factors explaining 87% of variance (37%, 23%, 15% and 12% resp). Factor 1 loaded on to eight respondents (5 male, 4SS, 3JS and 1IT). Within this factor, respondents preferred a CDSS providing recommendations suggesting some form of underlying reasoning by the system (AI-like decision support) over a CDSS that simply worked as a dictionary or encyclopedia (plain thesaurus functions). Transparency provided as information on why a suggestion was given by the CDSS was appreciated by all respondents loading on this factor. However, respondents favored statements in which the final decision was left in their hands. Factor 2 loaded on six respondents (5 male, 1IT, 3SS, 1JS), and was thus highly driven by male respondents. Personal contribution to the handling of the case was considered very important.

Factor 3 loaded to six respondents (3 female, 1SS, 3JS, 2IT). These respondents attributed less relevance to the AI-like support. Factor 4 loaded only to three respondents (2 female, 1SS, 2IT) and differed from factor 1 as it appreciated statement 4A, which describes a simple automated literature search. Statement 4G, providing a diagnostic suggestion and management proposal while still allowing a contribution by the physician, ranked positive in all factors.

### Qsort: thematic analysis of thinking aloud during surveys

**Fig. 1:**
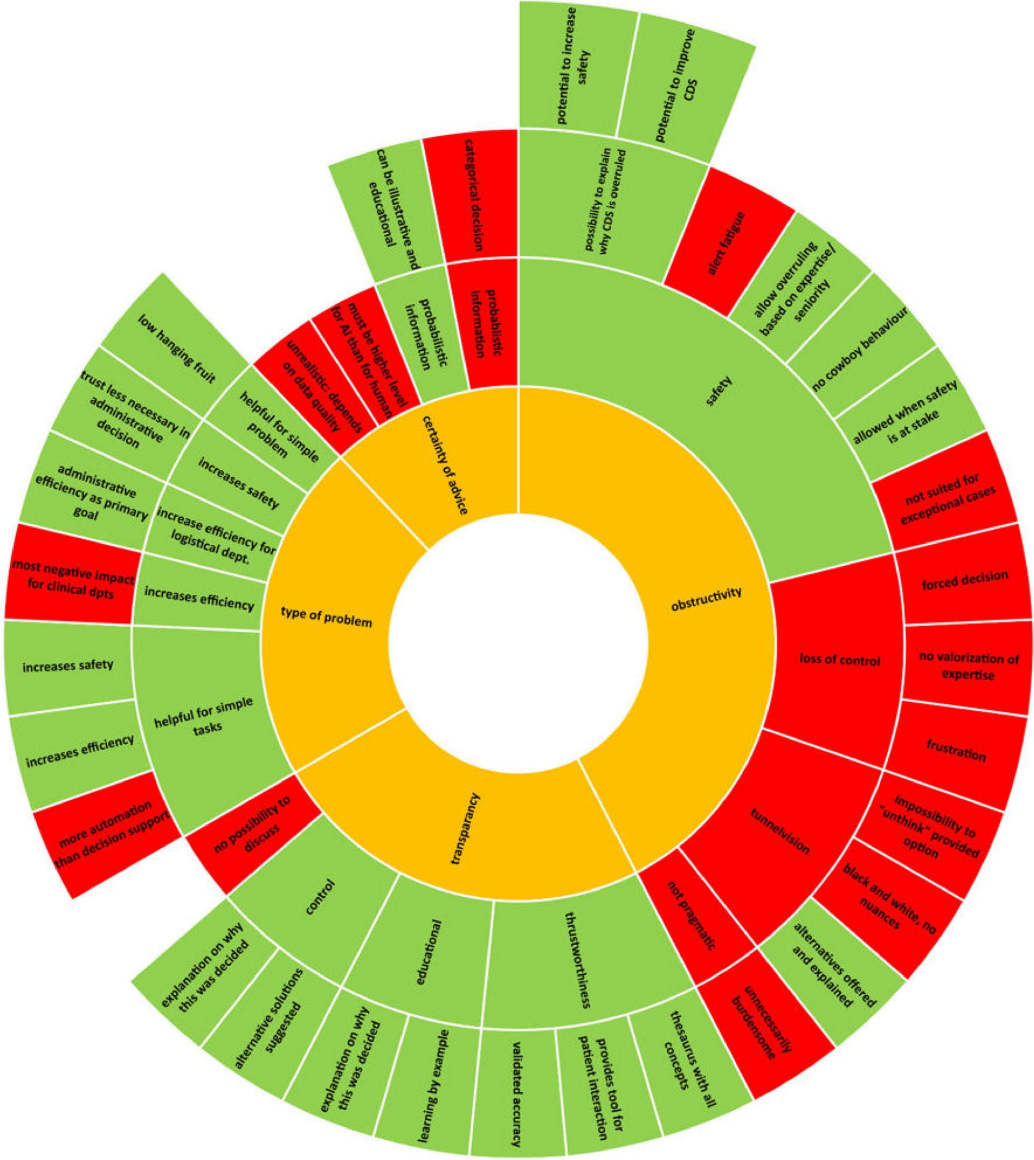
Thematic analysis. Inner side of the wheel: main themes (orange). More outward themes can be considered as subthemes of the more innerbound themes. Green color indicates a positively appreciated aspect, whereas red indicates a negatively appreciated theme

Figure 1 shows the subthemes related to the four themes that were hypothesized in the Qsort, and which emerged during the thinking aloud as derived from the field notes.

We identified four subthemes within the theme of transparency:

1. *Possibility for discussion and argumentation regarding the advice provided by the CDSS*. Several respondents indicated that it is impossible to reason or argue with a CDSS, whereas with other experts, peers or colleagues this interactive pro-con argumentation and discussion is an essential part of how the advice provided by the clinician comes about.

“the advice (of a CDSS) is absolute, and you cannot argue or ask for additional explanation as you would do with a colleague if you do not agree or have doubts” (R3)

“calling a colleague for advice is far more easy and effective” (R14)

“ [With people] you are able to ask why they suggested something, they are able to **give arguments** for their position and I am able to **react to those arguments**. But here [with the AI] it ends with a suggestion. ” (R1).

(2) *Feeling in control*. This was mainly associated with a good explanation of the underlying reasoning of the CDSS, or when different alternative options are offered and the physician has to make a choice.

“I like it that the system provides additional explanation on why it made this suggestion” (R9)

“Transparancy is good to create trust, and it provides control over the final decision” (R4)

“I feel more confident if I see that the system follows the same reasoning as I did” (R16)

“this is good as alternative solutions are offered, but I can make the final decision” (R11)

“The more information they get [from the AI], the more willing the physician will be to follow [the AI’s suggestion].” (R15)

(3) *Trustworthiness and accuracy of the advice*. Here some respondents indicated that there is a need for peer review of CDSS before it becomes implemented in the clinical workspace.

“Not only the system, but also medicine [as a field of study] has to have a certain level of accuracy [in order for these AI to function properly]. ” (R1)

“the quality and performance should be tested in a randomized trial” (R13)

“these (CDSS) should be peer reviewed, how else would I know their performance? ” (R12)

Other respondents suggested that trustworthiness can grow as the physician uses the system increasingly frequently and experiences the quality of the advice provided.

“if we start using them (CDSS), our confidence will grow as we will better understand what triggers the system and what makes it go astray” (R3)

(4) *Potential educational role of the CDSS*. It was suggested by several respondents that the advice as provided by the CDSS could be used to train more junior staff by flagging up alternatives.
  “popping up potential alternatives with their probability can be very instructive”

For the theme of expressing (un)certainty of the system regarding the correctness of its advice, we identified three subthemes:

1. Dependence on the quality of the data feeding the system:
  “even the best system (CDSS) is only as good as the quality of the data it is using, and we all know how many mistakes there are in medical records” (R4).

“The quality of the data the system is using will determine the quality of the system” (R2).

“yes, that system (a CDSS on infection control) is very helpful, but it is time consuming to get the data correctly in the system. ” (R17).

(2) Uncertainty and probabilistic nature of medicine as a specialty. According to this line of thought, the use of a CDSS is a potential pitfall, in view of the fact that medicine is too complex to be captured by a machine.
  “In medicine it is always important to doubt. … Our domain [medicine] is very hard to automate, because it is difficult to reduce it to well-defined patterns. With us there are way too many dimensions to take into account.” (R23).

It could however also be seen as a benefit, as a CDSS can be ideally suited to calculate and present probabilities.

(3) The notion that trustworthiness and accuracy should be higher for CDSS than for human decision makers.

For the theme of work flow interruption, we identified four subthemes:

The potential to increase safety was considered a benefit,

“[The CDSS obstructing certain actions] could be a good thing when you are dealing with cowboys, people who think they know it best. [Obstructing them via the CDSS] could be a good thing to protect people against themselves. ” (R20).

The other three subthemes, by contrast, were related to potential drawbacks of the CDSS: creation of tunnel vision, loss of human control, alert fatigue, and the added administrative burden (so-called ‘death by a thousand clicks’) [7].

“Do not bother me with unimportant news” (R5).

“I do not want 199 notifications” (R1).

Respondents seemed to make a difference in their appreciation of and trust in a CDSS according to the task being considered as administrative vs medical. CDSS were deemed to increase efficiency and safety of more simple, routine administrative tasks, whereas CDSS were considered unsuitable for medical tasks.

“[Unlike with medical decisions] I do trust the AI when it takes administrative decisions. Those do not look difficult to me. ” (R14).

[Administrative tasks] are trivial. They are very easy and should, obviously, be integrated [in the system]. (R6).

However, the added burden on clinicians to “feed the system” with data was also mentioned as a potential powerful distraction from their clinical tasks.

## Discussion

In summary, the current study suggests that avoidance of disruptive workflows and transparency of the underlying decision processes are important considerations when developing clinical decision support systems (CDSS) incorporated in Electronic Health Record (EHR) and Computerized Provider Order Entry systems (CPOE) systems. We conclude that not accounting for these aspects reduces the likelihood physicians will experience the CDSS as a useful tool, induces frustration and decreases job satisfaction. The study also found that obstructive interference is only acceptable when it increases safety, it is adjustable to user expertise level and/or allows deviations when explained by the end-user on why a deviation is desirable in this case. The latter can also contribute to education or improve the system’s decision making process. To the respondents in this study, transparency and explainability seem to boil down to providing sufficient clarification on the underlying determining factors and flowpaths within the CDSS, so that these can be cross-checked to their own knowledge, beliefs and convictions.

All current integrated CDSS can, next to offering an advice on demand of the user, also produce interruptive messages, i.e. messages that pop up automatically and interrupt the workflow planned by the user [26]. This can be intended to either improve safety, indicate logistical/practical issues or to guide the user to a change in behavior towards the solution presumed to be most optimal by the CDSS [5]. Such messages can be non-obstructive if the user can bypass it so she can continue the planned workflow, or obstructive when the message cannot be bypassed, and the user needs to change her originally planned workflow. Nearly all respondents in our Qsort study were strongly averse towards such interruptive messages, especially when also obstructive. Interruptive messaging has best been studied in drug-prescription applications [26]. Over half of the studies report a beneficial effect on prescriber behavior, but the impact on patient related outcomes is less clear [26]. Of note, such alerts have a rather high override rate [29, 30]. Obstructive alerts may also have unintended negative effects, such as a delay in prescribing off-label medication [31].

Our study indicates that interruptive messages are accepted by physicians if they add to patient safety, e.g. by blocking undesirable actions. Some indicated that obstructive messages can be allowed provided they allow overruling if the user has sufficient expertise (seniority) or when an explanation on why the decision was overruled is provided. Several studies suggest that an option to adapt alerts to local circumstances, expertise and workflows has a substantial positive impact on perceived user friendliness and task behavior [32, 33]. Such an approach allows to valorize the clinical expertise of the healthcare worker and restores their feeling of control. It also avoids the frustration of being forced to make decisions perceived to be inappropriate, for example in exceptional cases. This is an important point also for liability, as lay people may negatively judge physicians when they do not follow AI advice, except in non-standard settings [34]. In addition, some of our respondents suggested that explanation by experts on why they overruled the suggestions of the CDSS provide opportunities to improve the system.

The problem of alert fatigue is a well-established downside of interruptive messaging in CDSS [5, 14]. Nevertheless, our respondents only hinted at it indirectly as a theoretical problem. This can be due to the fact the current EHR system in our hospital does not already have a CDSS, so users were not yet confronted in their daily practice with this aspect. Some suggested to prioritize which messages should pop-up or not to avoid alert fatigue. Such tier systems reduce alert fatigue, and result in better uptake of the provided guidance [35]. It adds another argument for substantial local adaptability of EHR systems [4]. Automation complacency, operators not conducting sufficient (manual) checks themselves due to excessive thrust in the automated system, are the opposite of alert fatigue [21]. Respondents indicated people are most sensible to miss incorrect diagnoses of the CDSS if it is reliable most of the time, when the case and/or the advice appear as “near normal” and when they are overloaded with alternative tasks or tired. In relation to this, there was concern amongst the respondents that implementation of CDSS might lead to deskilling, or to delegation of tasks to staff with lower education, resulting in dangerous situations when the CDSS fails or when human interference is needed, such as exceptional cases.

Different facets of tunnel vision as another type of CDSS rigidity were brought up. First, once a suggestion is provided by the CDSS, this cannot simply be ignored. The physician needs to consider the suggestion, and reflect on it, at least internally. This might lead to unnecessary additional tests and tasks being done to be on the safe side. Second, there is little possibility to introduce a scale of gray in CDSS systems. Third, respondents identified the risk of anchoring bias [36, 37], i.e. looking for facts that confirm the suggestion of the CDSS whilst ignoring facts that don’t fit. Of note, physicians often fail to ignore inaccurate advice, regardless of it coming from another human or from an AI [38]. This effect is mitigated by the expertise level of the user [20], which would again support approaches in which suggestions of the CDSS can be overruled by more senior staff.

Some respondents indicated the CDSS might come up with useful suggestions they would not have considered themselves, thereby breaking their own internal mental tunnel vision [39]. Using a CDSS might also diminish confirmation bias, the process by which physicians believe data that support their own beliefs and dismiss those that refute their own intuition [39].

Our data also suggest that the type of task the CDSS takes on makes an impact on the acceptance of the generated advice. A CDSS is substantially better accepted for tasks considered to be administrative or easy, whereas there is much more reluctance to use CDSS for tasks considered to be more intellectual or complex. Administrative tasks can be thought of as CPOEs, or electronic medication prescription. Some authors distinguish systems only used for these tasks from true CDSS, whereas others don’t [5]. It might be that CDSS support in administrative tasks is more transparent as these are often rule based, whereas CDSS systems used for clinical/intellectual tasks tend be more based on machine learning and are thus less transparent. In general, implementation of CDSS for administrative or simple tasks is considered to be promising to increase efficiency [40]. Some raise concern this increased efficiency will be used to increase workload or reduce contact time with patients [10]. Others mention that the gain in efficiency is mostly for administrative and logistical departments, whereas the actual data input that enables this efficiency gain is an extra burden for the clinicians [4, 5]. It would also be interesting to know whether appreciation of CDSS is different for recommendations concerning primarily diagnostic tasks vs intervention and management oriented tasks. Unfortunately, the design of the current vignettes does not allow for that question to be answered, as this aspect was not elicited by any of the cases, hence insufficient data are available to draw any firm conclusions on this intriguing question.

Nearly all respondents highlighted that uncertainty is an essential aspect of clinical practice [41]. Accordingly, they expect the CDSS to provide a representation of how certain it is the provided advice is correct. There appears to be no clear preference for verbal vs numeric indication of that certainty amongst our respondents. Some argue that a verbal indication (might, likely, probably) is vague and not very useful. Most agreed that providing no grading of certainty automatically suggested that the CDSS was absolutely sure about the advice. There was also a general conviction amongst our respondents that the standards for certainty of advice should be higher for CDSS than for human providers. This request can either be explained by the absence of opportunity to discuss the provided advice, or by a lack of transparency.

Transparency and explainability are key issues in CDSS [42, 43]. There is however a lot of debate on what exactly is understood by these constructs, and what is essential to achieve them [44]. For our respondents, understanding the determining factors or the flow of arguments of the CDSS was considered crucial to create confidence in the advice. Trustworthiness was considered to be higher if the accuracy of the CDSS was validated in a clinical trial.

Providing a thesaurus with exact meaning of the terminology used by the CDSS was also considered necessary to enhance semantic transparency [45]. Using different concepts for the same disease, or different definitions for the same concept results in confusion e.g. in what exactly is predicted by prediction models [46]. Remarkably, merely providing an explanation was sufficient for most respondents, irrespective of the epistemic correctness of such explanation, as it provided a means of control. Others stressed that such explanations could be educative as they allowed “learning by example”.

In conclusion, acceptance of a CDSS incorporated in EHR/CPOE is improved by ensuring a swift integration into the workflow, with a system of options tailored to the expertise of the users to overrule the system. Transparency on the underlying processes is essential to gain trust. This perceived trust can be further enhanced by providing proof of the accuracy of the CDSS, either by personal experience or in randomized trials.

## Supplementary Information

**Additional file 1.** Qsort vignettes.

**Additional file 2.** Factor Scores with Corresponding Ranks.

**Additional file 3.** Factor Matrix with Defining Sorts Flagged.

**Additional file 4.** Factor Q-sort Values for Statements sorted by Consensus vs. Disagreement.

**Additional file 5.** Factor Q-sort Values for Statements sorted by Consensus vs. Disagreement.

## Data Availability

All data are supplied as supplementary materials. The precise content of the vignettes is available as appendix 1: Qsort vignettes; The data and analysis of the Qsort are available as dataset 1–4: Qsort analysis.
