# Supplementary material for: An exploration of expectations and perceptions of practicing physicians on the implementation of computerized clinical decision support systems using a Qsort approach
Source: BMC Med Inform Decis Mak. 2022 Jul 16;22:185. doi: 10.1186/s12911-022-01933-3 (PMC9288707; doi:10.1186/s12911-022-01933-3)
Supplement: Supplementary file 2 — Additional file 2. Factor Scores with Corresponding Ranks. [file 12911_2022_1933_MOESM2_ESM.pdf]

Factor Scores with Corresponding Ranks

Statement	Sta	Statement	factor 1 Z-score	factor 1 Rank	factor 2 Z-score	factor 2 Rank	factor 3 Z-score	factor 3 Rank	factor 4 Z-score
1	ya	1	-1,42	6	-1,45	6	0,16	3	0
2	yb	2	-0,71	5	-0,13	3	-1,49	6	0,71
3	yc	3	0,74	2	0,73	2	-0,75	5	-0,71
4	yd	4	0	3	-0,45	5	0,91	2	-1,41
5	ye	5	0	4	1,45	1	1,17	1	0
6	yf	6	1,39	1	-0,15	4	0	4	1,41

factor 4
Rank
3
2
5
6
4
1
