# Supplementary material for: An exploration of expectations and perceptions of practicing physicians on the implementation of computerized clinical decision support systems using a Qsort approach
Source: BMC Med Inform Decis Mak. 2022 Jul 16;22:185. doi: 10.1186/s12911-022-01933-3 (PMC9288707; doi:10.1186/s12911-022-01933-3)
Supplement: Supplementary file 3 — Additional file 3. Factor Matrix with Defining Sorts Flagged. [file 12911_2022_1933_MOESM3_ESM.pdf]

Factor Matrix with Defining Sorts Flagged

| Part.No. | | Q sort | Factor 1 | | Factor 2 |
|---|---|---|---|---|---|
| 1 | JVS | 11975234797 | 0,4033 | | 0,8402 |
| 2 | SSM | 11968113900 | 0,2932 | | 0,1626 |
| 3 | SSV | 11975200718 | 0,6843 | | 0,3553 |
| 4 | TV | 11956930576 | 0,6333 | | 0,2641 |
| 5 | SSV | 11968106889 | 0,8837 | flagged | 0,136 |
| 6 | TM | 11956705202 | 0,2857 | | 0,4575 |
| 7 | SSM | 11975225931 | 0,6709 | flagged | 0,2177 |
| 8 | SSM | 11975220030 | 0,9175 | flagged | 0,0528 |
| 9 | JSV | 11956716610 | 0,5158 | | -0,1935 |
| 10 | TM | 11975214073 | 0,6235 | | 0,5384 |
| 11 | JSM | 11918449255 | 0,4325 | | 0,4607 |
| 12 | JSV | 11918396760 | 0,8837 | flagged | 0,136 |
| 13 | TV | 11918199449 | 0,8176 | flagged | 0,5212 |
| 14 | TV | 11915284036 | 0,8837 | flagged | 0,136 |
| 15 | TV | 11915006700 | 0,1643 | | 0,686 |
| 16 | JSM | 11914905085 | 0,8311 | flagged | 0,3585 |
| 17 | SSM | 11908288882 | 0,1143 | | 0,1997 |
| 18 | SSV | 11908262344 | 0,8311 | flagged | 0,3585 |
| 19 | SSM | 11908257068 | 0,8581 | flagged | 0,2587 |
| 20 | JSM | 11908240588 | 0,0519 | | 0,9219 |
| 21 | SSM | 11898993445 | 0,8311 | flagged | 0,3585 |
| 22 | JSV | 11898874630 | 0,0519 | | 0,9219 |
| 23 | TM | 11898725940 | 0,2123 | | 0,7625 |
| 24 | TV | 11956700320 | 0,8649 | flagged | 0,2753 |
| 25 | SSV | 11995897780 | 0,9175 | flagged | 0,0528 |
| 26 | SSM | 11995639151 | 0,7774 | flagged | -0,2046 |
| 27 | SSV | 11995452143 | 0,8311 | flagged | 0,3585 |
| 28 | SSV | 11995416498 | 0,8311 | flagged | 0,3585 |
| 29 | SSV | 11995375905 | 0,5317 | | -0,0418 |
| 30 | SSM | 11995365593 | 0,2797 | | 0,7686 |
| %Explained Variance | | | 44 | | 21 |

| | Factor 3 | | Factor 4 | |
|---|---|---|---|---|
| flagged | 0,3103 | | -0,1521 | |
| | 0,7064 | flagged | -0,0258 | |
| | 0,0509 | | 0,6282 | |
| | 0,6487 | | 0,1687 | |
| | 0,378 | | 0,0188 | |
| | 0,3781 | | 0,7423 | flagged |
| | 0,5412 | | -0,279 | |
| | 0,1773 | | 0,1718 | |
| | 0,7329 | flagged | 0,2531 | |
| | 0,4591 | | 0,1876 | |
| | 0,7208 | flagged | 0,1678 | |
| | 0,378 | | 0,0188 | |
| | 0,1272 | | -0,1366 | |
| | 0,378 | | 0,0188 | |
| flagged | 0,0972 | | 0,6124 | |
| | 0,3936 | | 0,0538 | |
| | 0,8772 | flagged | 0,2514 | |
| | 0,3936 | | 0,0538 | |
| | 0,1862 | | 0,3414 | |
| flagged | -0,0371 | | 0,2122 | |
| | 0,3936 | | 0,0538 | |
| flagged | -0,0371 | | 0,2122 | |
| flagged | 0,5721 | | -0,1719 | |
| | 0,1928 | | 0,2067 | |
| | 0,1773 | | 0,1718 | |
| | 0,1107 | | 0,2613 | |
| | 0,3936 | | 0,0538 | |
| | 0,3936 | | 0,0538 | |
| | 0,7352 | flagged | -0,0506 | |
| flagged | 0,2817 | | 0,1606 | |
| | 19 | | 7 | |
