# Supplementary material for: An exploration of expectations and perceptions of practicing physicians on the implementation of computerized clinical decision support systems using a Qsort approach
Source: BMC Med Inform Decis Mak. 2022 Jul 16;22:185. doi: 10.1186/s12911-022-01933-3 (PMC9288707; doi:10.1186/s12911-022-01933-3)
Supplement: Supplementary file 4 — Additional file 4. Factor Q-sort Values for Statements sorted by Consensus vs. Disagreement. [file 12911_2022_1933_MOESM4_ESM.pdf]

Factor Q-sort Values for Statements sorted by Consensus vs. Disagreement

Statement	Sta	factor 1	factor 2	factor 3	factor 4	Z-Score variance
6	wf	1	1	2	0	0,183
3	wc	0	0	1	-1	0,243
1	wa	-2	-2	0	-2	0,316
4	wd	-1	0	0	2	0,667
2	wb	2	-1	-1	1	0,915
5	we	0	2	-2	0	1,025
