# Supplementary material for: An exploration of expectations and perceptions of practicing physicians on the implementation of computerized clinical decision support systems using a Qsort approach
Source: BMC Med Inform Decis Mak. 2022 Jul 16;22:185. doi: 10.1186/s12911-022-01933-3 (PMC9288707; doi:10.1186/s12911-022-01933-3)
Supplement: Supplementary file 5 — Additional file 5. Factor Q-sort Values for Statements sorted by Consensus vs. Disagreement. [file 12911_2022_1933_MOESM5_ESM.pdf]

Factor Q-sort Values for Statements sorted by Consensus vs. Disagreement

Statement	Sta	factor 1	factor 2	factor 3	factor 4	Z-Score variance
7	pg	1	0	1	0	0,317
4	pd	0	0	-1	1	0,327
6	pf	2	0	2	1	0,353
3	pc	0	-1	-2	0	0,366
8	ph	0	2	-1	0	0,556
2	pb	-1	1	0	-2	0,569
9	pi	-1	1	1	-1	0,761
5	pe	1	-2	0	-1	0,835
1	pa	-2	-1	0	2	1,681
